# Response to “AI Literacy in Health‐Professions Education: Deepening the Case for Curricular Reform in the Philippines”

**DOI:** 10.1002/hsr2.71359

**Published:** 2025-10-21

**Authors:** Seyyedeh Fatemeh Mousavi Baigi, Masoumeh Sarbaz, Ali Darroudi, Khalil Kimiafar

**Affiliations:** ^1^ Department of Health Information Technology, School of Paramedical and Rehabilitation Sciences Mashhad University of Medical Sciences Mashhad Iran; ^2^ Student Research Committee Mashhad University of Medical Sciences Mashhad Iran

Dear Editor,

We would like to express our sincere appreciation to the esteemed authors of the recent Letter to the Editor [1] for their thoughtful and well‐considered response to our recent study [2], published in the esteemed Health Science Reports. It is truly encouraging to see that our findings have served as a springboard for deeper reflection on AI literacy and curricular reform in health professions education in the Philippines.

Their recommendation to move beyond fragmented exposure to AI tools toward a more structured, competency‐based educational approach strongly resonates with the needs we observed among Iranian students. In particular, the need to strengthen foundational understanding of AI and address the gap between students' optimism and their actual readiness for clinical application is an issue that, in our view, warrants focused attention in future curriculum planning.

The three‐tiered framework they proposed—comprising foundational knowledge, applied judgment, and data governance—presents a practical and context‐sensitive model tailored to the Philippine setting. We believe this model holds great promise as an inspiring foundation for similar curriculum designs in other low‐ and middle‐income countries, including Iran.

We are especially grateful for their attention to critical issues such as ethics, assessment redesign, and faculty development—areas that also emerged as systemic needs in our own study and previous investigations [3, 4, 5]. Their response further underscores the idea that AI education must be implemented in a phased, purposeful manner, aligned with national policies and accreditation standards.

Moreover, we highlight the need to account for the accelerating pace of technological and scientific advancements in this field. Educational strategies for AI must be designed with agility and adaptability in mind, accompanied by regular evaluation cycles and real‐time feedback mechanisms to ensure continued relevance and effectiveness.

We hope this scholarly exchange will serve as the beginning of a broader global and interdisciplinary dialogue on preparing healthcare systems and professionals for the era of artificial intelligence. We look forward to future research efforts that operationalize and evaluate such frameworks across diverse contexts.

## Author Contributions

**Seyyedeh Fatemeh Mousavi Baigi:** conceptualization, writing – original draft, writing – review and editing. **Masoumeh Sarbaz:** writing – review and editing, conceptualization. **Ali Darroudi:** conceptualization, writing – review and editing. **Khalil Kimiafar:** conceptualization, writing – review and editing.

## Conflicts of Interest

The authors declare no conflicts of interest.

## Transparency Statement

The corresponding author (Khalil Kimiafar) affirms that this manuscript is an honest, accurate, and transparent account of the study being reported; that no important aspects of the study have been omitted; and that any discrepancies from the study as planned and study registration is not applicable.

## Data Availability

The authors have nothing to report.
